# Comparison of different real-time PCR chemistries and their suitability for detection and quantification of genetically modified organisms

**DOI:** 10.1186/1472-6750-8-26

**Published:** 2008-03-06

**Authors:** Meti Buh Gašparič, Katarina Cankar, Jana Žel, Kristina Gruden

**Affiliations:** 1 Department of Biotechnology and Systems Biology, National Institute of Biology, Večna pot 111, SI-1000 Ljubljana, Slovenia; 2 Plant Breeding – Wageningen UR, Droevendaalsesteeg 1, 6708 PB Wageningen, The Netherlands

## Abstract

**Background:**

The real-time polymerase chain reaction is currently the method of choice for quantifying nucleic acids in different DNA based quantification applications. It is widely used also for detecting and quantifying genetically modified components in food and feed, predominantly employing TaqMan^® ^and SYBR^® ^Green real-time PCR chemistries. In our study four alternative chemistries: Lux™, Plexor™, Cycling Probe Technology and LNA^® ^were extensively evaluated and compared using TaqMan^® ^chemistry as a reference system.

**Results:**

Amplicons were designed on the maize invertase gene and the 5'-junction of inserted transgene and plant genomic DNA in MON 810 event. Real-time assays were subsequently compared for their efficiency in PCR amplification, limits of detection and quantification, repeatability and accuracy to test the performance of the assays. Additionally, the specificity of established assays was checked on various transgenic and non-transgenic plant species. The overall applicability of the designed assays was evaluated, adding practicability and costs issues to the performance characteristics.

**Conclusion:**

Although none of the chemistries significantly outperformed the others, there are certain characteristics that suggest that LNA^® ^technology is an alternative to TaqMan^® ^when designing assays for quantitative analysis. Because LNA^® ^probes are much shorter they might be especially appropriate when high specificity is required and where the design of a common TaqMan^® ^probe is difficult or even impossible due to sequence characteristics. Plexor™ on the other hand might be a method of choice for qualitative analysis when sensitivity, low cost and simplicity of use prevail.

## Background

Real-time quantitative PCR (Q-PCR) is the state-of-the-art technology for the quantification of nucleic acids, both in gene expression analysis and in routine DNA quantification, and is becoming ubiquitous in research and diagnostics of various fields. Because of its prominence, several new Q-PCR detection chemistries were developed, reaching approximately 20 different at present [1]. Of all TaqMan^® ^and/or SYBR^® ^Green chemistries are being the most widely used [2]. The reason could lie in the numerous data available on the performance of these two methods, while only a few comparisons of the performance of alternative chemistries have been published [3-5].

The detection of genetically modified (GM) components is a challenging application of Q-PCR since exact quantification and the ability to detect trace amounts of GM material in food matrices is required. Given that the labelling threshold for food and feed is ranging from 5% in Japan [6] to as low as 0.9% in the European Union [7], an accurate quantification method is essential. To achieve this the efficiency of the PCR reactions must be close to the optimal efficiency of 1 where each PCR target is doubled in a course of one PCR cycle [8]. Deviations from optimal amplification can crucially affect the result of quantification [9]. The assays need to be robust and simple for use in routine analyses and additionally highly specific, so as not to detect related plant species or non-targeted GM organisms (GMO).

In the field of GMO detection MGB, Molecular Beacon [3], Amplifluor^® ^[4] and Scorpion^® ^chemistries [5] have already been compared to TaqMan^® ^and SYBR^® ^Green performance. To supplement the available knowledge we tested additional four new technologies: Locked Nucleic Acid (LNA^®^) Probes (Sigma Proligo, Paris, France), Cycling Probe Technology (CPT) (Takara, Shiga, Japan), Light Upon eXtension (Lux™) Fluorogenic Primers (Invitrogen Corporation, Carlsbad, United States) and Plexor™ Technology (Promega, Madison, United States). Their performance was compared to TaqMan^® ^chemistry (Applied Biosystems, Foster City, USA).

TaqMan^®^, LNA^® ^and CPT are all based on the design of a probe located between the two PCR primers and labelled with a reporter on the 5' and a quencher on the 3'-end. Following hybridization of the TaqMan^® ^or LNA^® ^probe to its complementary sequence within the PCR target, the probe is degraded due to the 5' > 3' exonuclease activity of *Taq *DNA polymerase. This separates the reporter dye from the quencher and its fluorescence intensity increases. The LNA^® ^probe differs from TaqMan^® ^by inclusion of modified nucleotides – named locked nucleic acids. These nucleic acid analogues form methylene bridges after binding and lock the structure on the target DNA. The melting temperature of these probes is thereby significantly increased; hence they can be designed to be shorter [10,11]. The CPT probe in contrast includes a modified RNA nucleotide forming a RNA-DNA duplex after hybridization to the target. In the next step this duplex is recognized and cut by RNaseH, resulting in separation of the quencher from the reporter, accompanied by a fluorescence increase. In this case no exonuclease activity of *Taq *DNA polymerase is needed to get an increase in the signal [12].

Lux™ and Plexor™ technologies do not employ a probe but rather use fluorescent labelling of one primer instead. In Lux™ technology one of the primers is labelled with a fluorophore close to the 3'-end which is quenched by the hairpin structure of the primer. On formation of the PCR product, the fluorescence increases up to 8-fold due to extension of the hairpin structure [13]. Plexor™ technology differs from the other chemistries in its strong fluorescence signal at the beginning of the reaction, which decreases proportionally to the increase of PCR products in the course of reaction. One of the primers contains a synthetic base, isocytosine, linked to the fluorophore at the 5'-end. During the amplification step the iso-dGTP from the reaction solution is preferentially incorporated in the opposite DNA strand and, because linked to the quencher, the signal decreases after the binding [14]. Both Lux™ and Plexor™ technologies allow for dissociation curves to be analyzed, additionally monitoring the specificity of the product.

Detection systems were compared on MON 810 GM maize, known also as YieldGard^® ^(zip code: MON-00810-6). It is one of the main GMO crops cultivated worldwide and especially interesting for development of quantitative methods due to its cultivation in the EU [15].

## Results and Discussion

**Figure 1:**
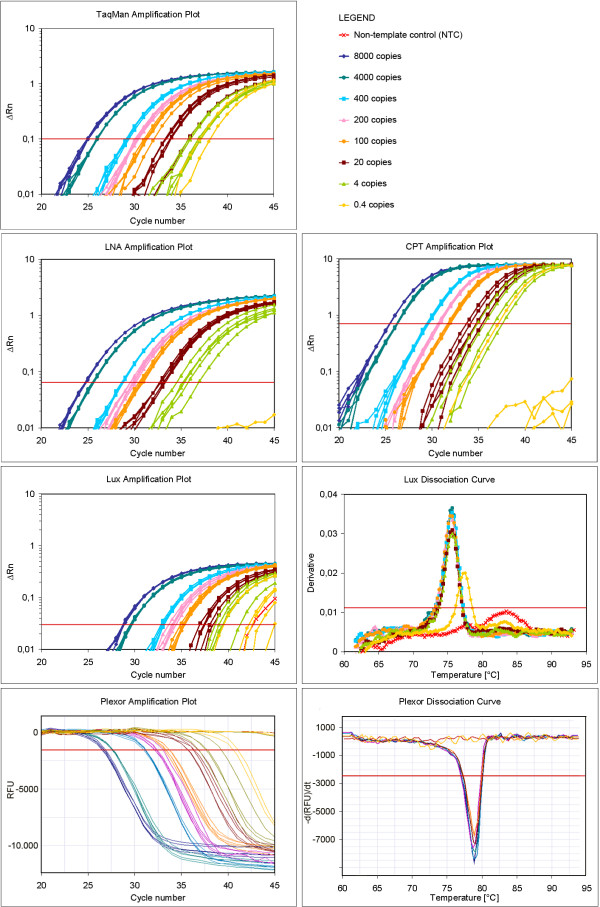
**Amplification plots and dissociation curves of Q-PCR reactions for different chemistries**. Amplification plots and dissociation curves for different detection chemistries are presented for real-time PCR reactions with 8000, 4000, 400, 200, 100, 20, 4 and 0.4 copies of MON 810 transgenic DNA. A signal for non-template control (NTC) is also shown. The fluorescence threshold is indicated by a horizontal line (ΔRn – normalized fluorescence; RFU – relative fluorescence unit).

**Figure 2:**
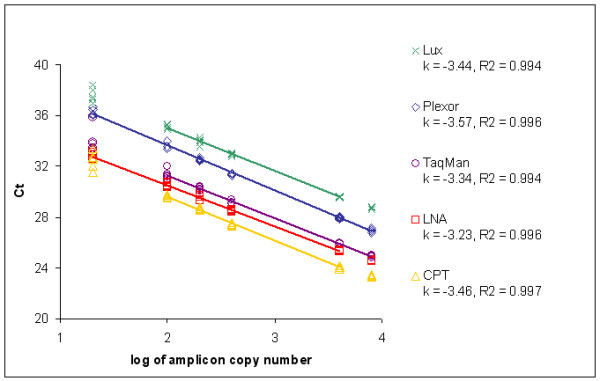
**PCR efficiency of different chemistries**. The Ct values for 8000, 4000, 400, 200, 100 and 20 copies of MON 810 transgenic DNA are plotted against the logarithm of the copy number. From the same data, slope (k) and determination coefficient R^2 ^were calculated for each of the chemistries. Values within the dynamic range are connected with a line (Ct-cycle threshold).

A total of 12 different assays were developed for detecting the invertase reference gene and the GM maize MON 810. The selected target sequence for the detection of MON 810 was the 5'-junction between the transformed organism's genome and inserted DNA which is event specific [16]. Two assays were developed for each of the probe based (LNA^® ^and CPT) and four for each of the primer based chemistries (Lux™ and Plexor™). Their performance was then evaluated according to parameters most important for routine analysis: limit of detection (LOD), dynamic range through limit of quantification (LOQ), amplification efficiency, repeatability and specificity. One of each Lux™ invertase assays and Lux™ MON 810 assays were excluded from further analysis due to low specificity and high LOQ, respectively. Similarly one of each Plexor™ invertase assays was excluded due to high LOQ while Plexor™ MON 810 assay was withdrawn because of an unavoidable primer dimer formation. These data are not shown in this manuscript. Methods' detailed performance was therefore studied on two assays per chemistry type, one on the invertase and the other on the 5'-junction of MON 810 (Table 1). Of the latter representative amplification plots with dissociation curves and standard curves for each of the chemistries are presented in Figure 1 and Figure 2, respectively.

**Table 1:** Primers and probes for Q-PCR analyses

**Detection method**	**Target**	**Amplicon (bp)**	**Primer/probe name**	**Oligo concentration (nM)**	**Sequence (5'-3')**^a^	**Position**^b^	**Reference**
TaqMan^®^	Ivr	79	Ivr_F	900	TGGCGGACGACGACTTGT	3376	[23]
			Ivr_R	900	AAAGTTTGGAGGCTGCCGT	3436	[23]
			Ivr_Taq_P	200	[FAM]-CGAGCAGACCGCCGTGTACTTCTACC-[TAMRA]	3395	[23]
	Mon	111	Mon_F	600	CCTTCATAACCTTCGCCCG	-87	[24]
			Mon_R	600	AATAAAGTGACAGATAGCTGGGCA	+5	[24]
			Mon_Taq_P	150	[FAM]-ACGAAGGACTCTAACGTTTAACATCCTTTGCCA-[TAMRA]	-30	[24]
LNA^®^	Ivr	79	Ivr_F	900	TGGCGGACGACGACTTGT	3376	[23]
			Ivr_R	900	AAAGTTTGGAGGCTGCCGT	3436	[23]
			Ivr_LNA_P	200	[FAM]-CGAGC+AG+ACCGCCGTG-[BHQ1]	3395	this study
	Mon	111	Mon_F	600	CCTTCATAACCTTCGCCCG	-87	[24]
			Mon_R	600	AATAAAGTGACAGATAGCTGGGCA	+5	[24]
			Mon_LNA_P	150	[FAM]-TTT+AA+C+AT+C+CTTTG+C+CA-[BHQ1]	-14	this study
CPT	Ivr	79	Ivr_F	600	TGGCGGACGACGACTTGT	3376	[23]
			Ivr_R	600	AAAGTTTGGAGGCTGCCGT	3436	[23]
			Ivr_CP_P	200	[FAM]-CT(G)CTCAAGGG-[TAMRA]	3420	this study
	Mon	112	Mon_CP_F	600	GAAGGACGAAGGACTCTAAC	-35	this study
			Mon_CP_R	600	GCAATGATGGCATTTGTAG	+58	this study
			Mon_CP_P	200	[FAM]-AT(G)GCAAAGGAT-[TAMRA]	-8	this study
Lux™	Ivr	84	Ivr_Lux_F	300	CGTGAGAATTTCCGTCTACTCG	2312	this study
			Ivr_Lux_R	300	cacggAAAGTGTTGTGCTTGGACCGt-[FAM]-G	2369	this study
	Mon	65	Mon_Lux_F	300	cgttagGAAGGACGAAGGACTCTAAc-[FAM]-G	-35	this study
			Mon_R	200	AATAAAGTGACAGATAGCTGGGCA	+8	this study
Plexor™	Ivr	99	Ivr_Plex_F	100	GCCGTGTACTTCTACCTGCTCA	3405	this study
			Ivr_Plex_R	100	[FAM]-iso-dC-GCATGGTCATAAGTCATAACATACATACCT	3478	this study
	Mon	134	Mon_F	200	CCTTCATAACCTTCGCCCG	-87	[24]
			Mon_Plex_R	200	[FAM]-iso-dC-CCTTTTCCACTATCTTCACAATAAAGTGAC	18	this study

^a ^+N denotes the LNA^® ^base; (G) stands for RNA nucleotide; lowercase letters for Lux™ indicate the sequence required for the hairpin structure and iso-dC is synthetic base isocytosine. ^b ^A position in the invertase gene or a distance in nucleotides between the 5'-junction and the 5'-end of the primer/probe.

### LOD, LOQ, dynamic range and efficiency of amplification

All the developed assays showed similar LOD and enabled detection of at least 20 copies of the target DNA (Table 2). LNA^®^, Lux™ and Plexor™ chemistries proved to be the most sensitive, as all of them detected 4 copies of DNA in one experimental system. The LOQ results on the other hand were more heterogeneous (Table 3). The highest LOQ was determined for the invertase amplicon for CPT and Lux™ chemistries where reliable quantification could be achieved only when 400 or 200 copies of the target DNA were present in the PCR reaction, respectively. The LOQ determined for these chemistries on the MON 810 amplicon was however comparable to other assays. The lowest LOQ was determined for both LNA^® ^assays, where as few as 20 copies of target DNA could be reliably quantified. From the statistical point of view however, less than 35 copies cannot be precisely and accurately quantified [17]. This discrepancy can be attributed to the inaccuracy of copy number estimation. The upper limit of the dynamic range was additionally monitored by measuring the PCR amplification at high target copy number per reaction. In none of the assays was PCR inhibition so severe as to completely inhibit PCR amplification, which would have resulted in absence of a fluorescent signal. However, at high target copy numbers, a decrease in amplification efficiency occurred in most of event specific amplicons. Plexor™ MON 810 amplicon was the least sensitive to inhibition at high copy numbers of all the chemistries tested, since no inhibition was detected on dilution series (Table 2).

**Table 2:** Average Ct values and coefficients of variation (Cv) for two dilution series per assay

	**Invertase**					**MON 810 5'-junction**				
		
**Investigated characteristic**	**TaqMan^®^**	**LNA^®^**	**CPT**	**Lux™**	**Plexor™**	**TaqMan^®^**	**LNA^®^**	**CPT**	**Lux™**	**Plexor™**
Detection limit (No. of copies)	4	20	20	20	4	4	4	20	4	20
Quantification limit (No. of copies)	100	100	400	200	100	100	20	100	100	100
Amplification efficiency (%)	95	97	93	90	91	97	101	98	93	86
Dynamic range (No. of copies)	100–40 000	100–40 000	400–40 000	200–40 000	100–40 000	100–4000	20–4000	100–4000	100–4000	100–8000
Repeatability in dynamic range, Cv (%)	7.5	10.6	13.7	10.4	12.7	10.3	9.6	4.9	11.3	12.7
PCR inhibition at the highest copy number (No. of inhibited cases/all cases)	0/2	0/2	0/2	0/2	0/2	1/2	2/2	2/2	2/2	0/2

**Table 3:** Performance of different chemistries, compared as LOD, LOQ, efficiency, dynamic range and repeatability

**No. of copies**	**TaqMan^®^**^c^		**LNA^®^**		**CPT**		**Lux™**		**Plexor™**	
**Invertase**										
40000	**24.07 (6)**	**23.84 (6)**	**23.15 (14)**	**23.00 (6)**	**24.74 (4)**	**25.13 (11)**	**26.76 (4)**	**26.69 (2)**	**24.41 (11)**	**24.57 (8)**
4000	**27.57 (6)**	**27.31 (3)**	**26.82 (9)**	**26.52 (8)**	**28.25 (13)**	**28.21 (13)**	**30.51 (11)**	**30.23 (8)**	**27.96 (11)**	**28.13 (7)**
400	**30.94 (10)**	**30.68 (8)**	**30.14 (19)**	**29.93 (9)**	**31.98 (13)**	**31.91 (23)**	**34.33 (15)**	**34.07 (9)**	**31.44 (11)**	**31.61 (14)**
200	**32.09 (6)**	**31.62 (3)**	**31.06 (8)**	**30.83 (7)**	**33.09 (17)**	32.16 (25)	**34.82 (12)**	**34.70 (22)**	**32.63 (5)**	**32.51 (21)**
100	**33.00 (15)**	**32.63 (13)**	**32.24 (18)**	**31.80 (8)**	33.25 (26)	33.75 (49)	35.71 (39)	35.59 (25)	**33.46 (24)**	**34.00 (14)**
20	35.85 (43)	**35.28 (15)**	**34.31 (19)**	34.16 (32)	38.89 (124)	4/5	3/5	3/5	35.72 (35)	35.90 (41)
4	4/5^a^	4/5	2/5	1/5	4/5	1/5	2/5	1/5	4/5	3/5
0.4	1/5 ^a^	2/5	0/5	1/5	1/5	0/5	0/5	0/5	1/5	0/5
										
**MON 810**										
8000	25.23 (2)^b^	24.95 (5)	24.82 (4)^b^	24.55 (3)^b^	23.38 (3)^b^	24.40 (3)^b^	28.78 (6)^b^	28.76 (6)^b^	**26.96 (9)**	**25.42 (11)**
4000	**26.00 (3)**	**25.92 (2)**	**25.41 (5)**	**25.34 (3)**	**24.11 (7)**	**25.17 (3)**	**29.59 (4)**	**29.59 (5)**	**27.94 (5)**	**26.58 (16)**
400	**29.41 (15)**	**29.14 (8)**	**28.56 (9)**	**28.53 (4)**	**27.42 (5)**	**28.38 (4)**	**32.82 (14)**	**32.95 (7)**	**31.37 (5)**	**30.49 (15)**
200	**30.57 (12)**	**30.24 (10)**	**29.66 (13)**	**29.57 (17)**	**28.67 (6)**	**29.44 (2)**	**34.23 (13)**	**33.98 (18)**	**32.53 (7)**	**31.60 (14)**
100	**31.54 (13)**	**31.38 (24)**	**30.81 (4)**	**30.53 (12)**	**29.63 (7)**	**30.43 (8)**	**35.33 (19)**	**35.14 (11)**	**33.71 (18)**	**32.65 (19)**
20	33.94 (31)	34.06 (47)	**33.19 (11)**	**32.74 (17)**	32.46 (44)	33.47 (26)	38.61 (73)	37.62 (35)	**36.25 (19)**	34.86 (28)
4	4/5	36.60 (44)	4/5	3/5	4/5	2/5	3/5	39.45 (50)	4/5	2/5
0.4	2/5	1/5	0/5	1/5	1/5	0/5	1/5	2/5	0/5	0/5

^a ^X/X; number of positives/number of experiments, Cv was not calculated when some of the parallels failed to amplify; ^b^inhibition was observed in these reactions as decrease in efficiency of amplification; ^c ^well established TaqMan^® ^based amplicons were analyzed in parallel as a reference system. Values within the dynamic range are shown in bold.

All the amplicons investigated showed satisfactory efficiency of amplification (90% ≤ E ≤ 110%), with the exception of the MON 810 amplicon for Plexor™ chemistry (Table 3). In this case an efficiency of 86% was obtained. Similarly, Lux™ chemistry, that is also based on fluorescently labelled primers, showed somewhat lower efficiency than the probe based chemistries.

The absolute cycle threshold (Ct) values at the same DNA concentration were compared at optimal baseline and threshold settings for all detection chemistries. The Ct values obtained for the newly designed LNA^®^, CPT and Plexor™ assays differed from those for the TaqMan^® ^assay by less then one Ct. However, the same amount of DNA was detected by Lux™ chemistry approximately 3 cycles later than by TaqMan^® ^chemistry, which indicates the difference in signal intensity.

### Specificity of the assays

The specificity of the maize endogenous gene invertase assays was confirmed by testing the amplification of DNA from the plant species *Nicotiana tabacum*, *Solanum lycopersicum, Capsicum annuum, Brassica oleracea *spp. *botrytis*, spp. *oleracea *and spp. *gongylodes, Triticum aestivum, Dianthus caryophyllus *and *Solanum tuberosum*. The cross-reactivity with DNA of Roundup Ready GM soybean, GM cotton and GM oilseed rapes RT73 and Topas 19/2 was also tested (Table 4). No cross-reactivity was detected with any of the DNA samples tested. Successful invertase amplification in non-transgenic maize DNA and GM maize Bt11, Bt176, TC1507, T25, MON 863, NK603 and GA21 was confirmed, as expected. The only exception was the CPT assay that detected *Brassica oleracea *spp. *gongylodes*, which might have resulted from probe's very short length (11 bp). It was however significantly less sensitive towards *Brassica oleracea *spp. g*ongylodes *DNA indicated by a 10 Ct difference at the same copy number. When checking the specificity of MON 810 assays, no cross-reactivity was observed with a mixed sample containing 8 different GM maize lines (Table 4). Only in the case of the Plexor™ chemistry based MON 810 assay a weak positive signal was detected, observed at only 0.2 Ct under the non-template control (NTC). Therefore this assay was further tested on the set of specific GM maize lines and it was shown that it cross-reacts with Bt176 (sensitivity ca 20-fold lower, results not shown).

**Table 4:** Analysis of invertase and MON 810 assay specificity for developed assays employing different chemistries

		**Result for maize invertase assay**				**Result for MON 810 assay**			
			
**Common name**	**Scientific name/GMO ZIP code**	**LNA^®^**	**CPT**	**Lux™**	**Plexor™**	**LNA^®^**	**CPT**	**Lux™**	**Plexor™**
**Nontransgenic plants**									
maize	*Zea mays*	+	+	+	+	NT	NT	NT	NT
tobacco	*Nicotiana tabacum*	-	-	-	-	NT	NT	NT	NT
tomato	*Solanum lycopersicum*	-	-	-	-	NT	NT	NT	NT
sweet pepper	*Capsicum annuum*	-	-	-	-	NT	NT	NT	NT
cauliflower	*Brassica oleracea *spp. *botrytis*	-	-	-	-	NT	NT	NT	NT
cabbage	*Brassica oleracea *spp. *oleracea*	-	-	-	-	NT	NT	NT	NT
kohlrabi	*Brassica oleracea *spp. *gongylodes*	-	+	-	-	NT	NT	NT	NT
wheat	*Triticum aestivum*	-	-	-	-	NT	NT	NT	NT
carnation	*Dianthus caryophyllus*	-	-	-	-	NT	NT	NT	NT
potato	*Solanum tuberosum*	-	-	-	-	NT	NT	NT	NT
									
**Transgenic plants**									
YieldGard^® ^maize	MON-00810-6 (MON 810)	+	+	+	+	+	+	+	+
MON 863 maize	MON-00863-5 (MON 863)	+	+	+	+	-	-	-	-
Bt11 sweet maize	SYN-BT011-1 (Bt11)	+	+	+	+	-	-	-	-
NaturGard™ maize	SYN-EV176-9 (Bt176)	+	+	+	+	-	-	-	+
Roundup Ready^® ^maize	MON-00021-9 (GA21)	+	+	+	+	-	-	-	-
Liberty-Link™ maize	ACS-ZM003-2 (T25)	+	+	+	+	-	-	-	-
Roundup Ready^® ^maize	MON-00603-6 (NK603)	+	+	+	+	-	-	-	-
Herculex^® ^I maize	DAS-01507-1 (TC1507)	+	+	+	+	-	-	-	-
StarLink™ maize	ACS-ZM004-3 (CBH351)	NT	NT	NT	NT	-	-	-	-
Roundup Ready^® ^soybean	MON-04032-6 (GTS 40-3-2)	-	-	-	-	-	-	-	-
Topas 19/2 oilseed rape	ACS-BN007-1	-	-	-	-	-	-	-	-
Westar Roundup Ready^® ^oilseed rape	MON-00073-7 (RT73)	-	-	-	-	-	-	-	-

NT – not tested.

### Repeatability of the assays

Repeatability of Q-PCR reactions was assessed by calculating the coefficient of variation (Cv) of parallel reactions within the dynamic range of the assay. The results for different assays show variability of 4.9 to 13.7% (Table 2 and Table 3). All the chemistries showed similar repeatability at high target copy numbers per reaction, they however performed differently at low copy numbers. While primer based chemistries had higher Cv values, LNA^® ^and one of the CPT amplicons showed better repeatability, being precise even below 100 DNA copies.

### Comparison of trueness of different methods

The trueness for the different assays was compared on samples of 100% and 0.29% MON 810 to check the performance of methods at the limits of the dynamic range. Results for the 100% MON 810 sample show that only TaqMan^® ^and LNA^® ^chemistries gave results within the 33% interval from the true value (Table 5). The GM percentage estimation obtained by Plexor™ and CPT chemistries differed substantially from the true result. Lux™ chemistry did not perform well considering either trueness or precision.

**Table 5:** Accuracy of quantification performed by different chemistries

	**100% MON 810**	**0.29% MON 810**	
		
**Detection chemistry**	**determined GMO content (%)**	**determined GMO content (%)**	**z-value**
TaqMan^®^^a^	73 ± 6	0.24 ± 0.15	0.19
LNA^®^	95 ± 10	0.12 ± 0.05	-0.91
CPT	51 ± 16	0.28 ± 0.09	0.43
Lux™	65 ± 34	0.45 ± 0.45	0.37
Plexor™	50 ± 6	0.27 ± 0.09	1.18

^a ^Well established TaqMan^® ^based amplicons were analyzed in parallel as a reference system.

Z-scores were calculated to evaluate trueness of quantitative analysis of USDA Proficiency test sample (0.29% sample), as if we would participate in December 2004 round of Proficiency testing, taking into account the results from other participating laboratories. For all chemistries calculated z-score was below 1 and therefore showing satisfactory trueness range. It should be borne in mind however that, at GM content below 1%, results often differ more from the true value than at the content above 1%. This can also be seen from the results of validation reports of methods (gmo-crl.jrc.it). Taking that into account, all the estimates are satisfactory except maybe the one with Lux™ chemistry showing high measurement uncertainty. Even at low GM content the precision of LNA^® ^and Plexor™ chemistries were best. Nevertheless these results are only an indication of the assays' trueness, and more studies should be performed to increase the reliability of these data.

In another publication, the first useful model of Lux™ chemistry was reported for event-specific detection and quantification of RRS [18]. The obtained values of LOD, LOQ and range of quantification were similar to ours, although primer dimer formation was observed at much higher DNA concentrations (i.e. 1000 copies) than in our case. In accuracy studies however better trueness of the method was reported while repeatability was quite low in their experiments as well.

### Practicability of different assays

High performance is essential to obtain a method sufficiently robust for use in different laboratories. For routine analysis some other parameters of the method also have to be considered, i.e. time needed to introduce the method into the laboratory and time required to perform the analysis. Last but not least the costs of the assay are very important (Table 6). Yet another parameter which could be critical for wider acceptance of the chemistry is how sequence dependant is the ease of method design.

**Table 6:** Comparison of general applicability and practicability of the designed assays

	**Modified probes**			**Modified primers**	
		
**Investigated characteristic**	**TaqMan^®^**^a^	**LNA^®^**	**CPT**	**Lux™**	**Plexor™**
Labour intensity to design	low	low	middle	high	middle
Primer/probe design	software	on demand	on demand	software	software
Redesigns required (No. of unsuccessful/successful designs)	-	0/2	1/2	3/4	1/4
Price to establish a new system	high	high	very high	middle	middle
Price for 100 × 10 μl reactions in Euros^a^	46	48	185	50	42
Run duration (minutes)	120	120	84	144	87
Labour intensity to analyze	low	low	low	middle	middle

^a ^Well established TaqMan^® ^based amplicons were analyzed in parallel as a reference system. ^b ^Estimated costs include PCR reagent, primers and probes but exclude plastics and optical covers since they are the same for all tests. Prices are stated for Slovenia, European Union, and may differ in other countries.

When used to working with TaqMan^® ^chemistry, switching to LNA^® ^is the easiest option. The probe is designed by the provider, while the same primers, master mix and the PCR protocol can be kept. Omitting a probe should in general simplify new assay design, but Lux™' specific design requirements were not always easy to meet. Consequently, efficient Lux™ amplicon design required several repeated trials. For both primer based chemistries, extensive optimization of primer concentrations was required to minimize the formation of primer dimers. Nonetheless dimers still appeared in some reactions when DNA concentration was low (i.e. below 100 copies), more often for Lux™ chemistry than for Plexor™, as Plexor™ master mix is optimized to minimize dimer formation.

Plexor™ and Lux™ chemistries were the least expensive systems to establish, as there was no need to order an expensive probe with each design trial. Omitting the probe can lower the price of routine analyses as well, although the influence of primers or probes on the overall reaction price is minor. The assay price depends more on the master mix, therefore price differences between TaqMan^®^, LNA^®^, Lux™ and Plexor™ chemistries do not exceed 15%. The only exception is the very high price of CPT assays, which would exclude this chemistry from use in routine analyses. Plexor™ seems appropriate also due to shorter experimental run duration.

What a potential user of Plexor™ might dislike is that DNA has to be prepared in a special buffer and that, after the run, special (publicly available) software has to be used for data analysis; however we have not experienced any problems while introducing this alternative system.

## Conclusion

A growing number of Q-PCR detection chemistries is available to detection laboratories. In this paper four different chemistries: LNA^®^, CPT, Lux™ and Plexor™ were compared to TaqMan^® ^for GMO detection and quantification. Their advantages and drawbacks were highlighted and their influence on the final results of quantification was demonstrated. It should however be taken into account that both TaqMan^® ^methods were already proven to be robust and reliable through use in routine GMO detection, while the alternative methods were developed and optimized only to the degree described in this paper.

Differences in performance of the methods were observed in regard to quantification and detection limits, amplification efficiency, specificity, trueness and practicability. These characteristics should be considered when designing new quantification assays, especially for routine analyses.

The criteria by which the methods are chosen must be carefully considered. In the case of GMO, detection methods need to perform either rapid and economically feasible screening for the presence of GMOs or exact quantification of GMOs in plant derived materials. High sensitivity is important for the detection of highly degraded DNA in processed food products. With the increasing number of GMOs in the market, the number of analyses performed per sample, and consequently the costs of analyses, increase. Therefore the practicability of the assays, in terms of duration of the analysis and time investment for the design and data analysis, has to be considered.

Of the probe based methods, LNA^® ^chemistry is the most promising, with excellent quantification limits and efficiency (Table 3). Very good repeatability, even for low copy numbers, is reflected in high precision and accuracy of measurements (Table 5). LNA^® ^methods can be easily transferred from the widely used and certified TaqMan^® ^methods should this prove beneficial for some applications. Because LNA^® ^probes are much shorter they could be especially appropriate where high specificity is needed (e.g. only one nucleotide difference in the sequence). They are also likely to be used where the sequences are such that the design of a common TaqMan^® ^probe is difficult or even impossible, for example in detecting junctions between GM insert and plant DNA.

Due to some performance characteristics (Table 3) it is not likely that Lux™ or Plexor™ chemistries would replace the probe based chemistries in the quantification of GMO content, especially for samples with multiple ingredients. With the probe absent, a perfect specificity is even harder to achieve, which also showed as slight crossreactiveness in one of Plexor™ designs (Table 4). Plexor™ chemistry however performed well when considering LOD. In addition it was the most robust against inhibitory substances of all the chemistries tested and proved practical for routine use. We believe that with additional effort put in design of specific primers Plexor™ technology provides an appropriate and affordable approach for qualitative analysis.

TaqMan^®^, MGB, Molecular Beacon and SYBR^® ^Green based detection methods were similarly compared for detection and quantification of RRS [3] and none of the approaches appeared significantly better. In our experimental comparison however, results suggest that probe based TaqMan^® ^and LNA^® ^technologies are best for quantitative analysis. Primer based Plexor™ on the other hand could be the method of choice for qualitative analysis if appropriately designed to assure specificity of the method.

## Methods

### Plant Materials

Certified reference material containing 5% MON 810 GM maize was purchased from the Institute for Reference Materials and Measurements (IRMM, Geel, Belgium). Seeds of MON 810, variety Campero, were provided by Institut de Biologia Molecular de Barcelona, Consorci CSIC-IRTA (Barcelona, Spain). Specificity studies for invertase were performed on 11 plant species. Seeds of *Nicotiana tabacum *L. (cultivated tobacco), var. White Barley, *Solanum lycopersicum *L. (tomato) var. Money Maker and *Capsicum annuum *L. (sweet pepper) were from the seed collection of the National Institute of Biology (NIB, Ljubljana, Slovenia). Seeds of *Brassica oleracea *L. spp. *botrytis *L. (cauliflower), *Brassica oleracea *L. spp. *oleracea *L. (cabbage) var. Brunswick, *Brassica oleracea *L. spp. *gongylodes *L. (kohlrabi), *Triticum aestivum *L. var. Soissons (wheat) and *Dianthus caryophyllus *L. (carnation) were purchased from the local seed company. Seeds were planted and leaf material of individual plants was used for DNA isolation. Non-GM maize (*Zea mays *L.) was obtained as a sample from routine GMO analysis and confirmed to be non-transgenic by screening for the presence of p35S promoter and tNOS terminator [19,20]. Leaves of *Solanum tuberosum *L. var. Desiree (potato) were taken from the plant collection of NIB. Certified reference materials for Bt11, Bt176, TC1507, MON 863, NK603 and GA21 GM maize and Roundup Ready^® ^soybean (*Glycine max *L. Merr.) (RRS) were purchased from IRMM. GM maize T25 was obtained from Aventis CropScience NV (Ghent, Belgium). Isolated DNA from GM maize events NK603, GA21 and CBH351 was purchased from Fluka (Buchs, Switzerland). GM oilseed rape RT73 (*Brassica napus *L.) was purchased from AOCS (The American Oil Chemists' Society, Champaign, USA) and GM oilseed rape Topas 19/2 was provided by Bayer CropScience (Monheim am Rhein, Germany). Samples with known concentrations of different GM events were obtained from USDA/GIPSA Proficiency Program (United States Department of Agriculture, Grain Inspection, Packers and Stockyards Administration, Washington DC, USA). The composition of the samples was as follows: 1C 2003/5 (2.5% Bt11, 1% Bt176, 2.3% GA21, 0.5% T25, 1.1% NK603 and 0.1% CBH351), C 2005/14 (0.06% Bt11, 0.03% TC1507, 1.3% T25 and 0.7% NK603) and 2C 2004/4 (0.4% MON 810, 0.4% T25, 0.4% CBH351, 0.4% Bt176, 0.1% TC1507 and 0.8% MON 863). Although the sample 2C 2004/4 was prepared to contain 0.4% (w/w) of MON 810 maize, the reported mean value of 23 laboratories involved in the proficiency scheme was 0.29% of MON 810 [21], therefore this value was taken as a reference for our experiments.

### DNA Isolation and quantification

Maize grain was homogenized by an Ultra Centrifugal Mill ZM100 (Retch, Haan, Germany) to obtain particles of less than 1 mm in diameter. Leaf material was ground by a micropestle in microcentrifuge tubes immersed in liquid nitrogen. DNA from powdered grain material, 0.29% MON 810 sample and potato leaf DNA were purified by NucleoSpin^® ^Food Kit (Macherey-Nagel, Düren, Germany) as described by the manufacturer. Genomic DNA from other leaf material was extracted using the DNeasy Plant Mini kit (Qiagen, Valencia, CA), except MON 810 DNA, which was purified by Magnetic Wizard^® ^Food kit (Promega, Madison, USA). The concentration of isolated DNA was determined by measuring the fluorescence of PicoGreen^® ^dye (Invitrogen) at 353 nm in a GENios fluorometer (Tecan, Crailsheim, Germany). The genome copy number of DNA isolates was determined by the Q-PCR TaqMan^® ^invertase assay, using known copy number of 5% MON 810 certified reference material for the construction of the standard curve. The 5% MON 810 standard reference material has been diluted to contain approximately 100, 33, 11, 3.7 and 1.2 ng of DNA which corresponds to approximately 36000, 12000, 4000, 1300 and 440 maize genome copies per reaction on the basis of the maize genome size [22]. Copy number was calculated by interpolation of Ct values generated in a standard regression curve.

Quality of all extracted DNA was checked through measurement of efficiency of amplification using TaqMan^® ^invertase and MON 810 assay [9]. The difference in efficiency between all DNA extracts proved to be less than 15% showing that they can all be used in quantitative analysis.

### Assay design and optimisation

Primers and probes were designed on the maize invertase as a reference gene and on the 5'-junction of MON 810 event. Gene sequences used for primer design were obtained from the public database of the National Center for Biotechnology Information (NCBI) with accession numbers U16123 and AF434709, respectively. For TaqMan^® ^based detection, previously published primers and probes for invertase gene [23] and 5'-junction of MON 810 [24] were used, synthesized by Applied Biosystems. The same primers were used for the LNA^® ^chemistry based assay, while specific LNA^® ^probes were designed and synthesized by Sigma, Proligo. For CPT invertase assay the same primer pair was used, while MON 810 primers and both probes were designed by Takara. The probes were synthesized by Takara and the primers by MWG Biotech AG (München, Germany). For the design of Lux™ and Plexor™ assays, online primer design software D-Lux™ Designer (Invitrogen) and Plexor™ Primer Design Software (Promega) were used, and the primers were synthesized by Invitrogen and IDT (Integrated DNA Technologies, Coralville, IA), respectively. The specificity of the designed primers was predicted *in silico*, using BLAST analysis of NCBI public database. FAM fluorescent dye (6-carboxyfluorescein) attached to the 5'-end of oligonucleotides was used as a reporter dye, while two different quenchers were used at the 3'-end of the probes: TAMRA (6-carboxytetramethylrhodamine) for TaqMan^® ^and CPT, and BHQ1 (Black Hole Quencher) for LNA^® ^technology (Table 1).

The concentrations of all primers and probes were optimized prior to method performance characterization. Three different concentrations were examined – the one recommended from the producer, one below and one above this concentration, using Q-PCR conditions described below. For further analysis the combination with the highest signal intensity at the lowest appearance of primer dimers was selected. In addition, the concentration of the passive reference dye ROX was optimized for all alternative chemistries except Plexor™ (results not shown).

TaqMan^® ^amplicons for invertase [23] and 5'-junction of plant genomic DNA and MON 810 insert [24] used in our lab for routine GMO detection were analyzed in parallel as a reference system.

### Q-PCR

The Q-PCRs were run on the ABI PRISM^® ^7900 HT sequence detection system (Applied Biosystems). Reactions were performed in 10 μl reaction mixture volumes with cycling conditions set to the recommended protocols of the manufacturer. TaqMan^® ^and LNA^® ^assays contained 1 × TaqMan^® ^Universal PCR Master Mix (Applied Biosystems) including a passive reference dye ROX and uracil N-glycosylase (UNG) to prevent carryover contamination. PCR cycling conditions were set to 2 min at 50°C and 10 min at 95°C followed by 45 cycles of 15 s at 95°C and 1 min at 60°C. For CPT assays CycleavePCR^® ^Core kit (Takara) was used with 0.1 μl ROX dye (Invitrogen) added to each reaction. The PCR program consisted of 30 s at 95°C followed by 45 cycles of 5 s at 95°C, 20 s at 55°C and 15 s at 72°C. Lux™ assays contained 1 × Platinum^® ^Quantitative PCR SuperMix-UDG (Invitrogen) with 0.4 μl ROX dye (Invitrogen) added per reaction. The PCR cycling program consisted of 2 min at 50°C and 2 min at 95°C followed by 45 cycles of 15 s at 95°C, 30 s at 55°C and 30 s at 72°C. Plexor™ assays were used with 1 × Plexor™ Master Mix (Promega). The PCR program consisted of 2 min at 95°C followed by 45 cycles of 5 s at 95°C and 35 s at 60°C. For Lux™ and Plexor™ chemistries a dissociation stage was included after the PCR with 15 s at 95°C, 15 s at 60°C and 15 s at 95°C, with a temperature ramp rate at 2%. Data were analyzed using the SDS 2.2.2 software (Applied Biosystems) for all the chemistries except Plexor™ where raw data was exported to Plexor™ Analysis Software (Promega). Ct values were determined with automatically set baseline and manually adjusted fluorescence threshold. After being exported further data analysis was performed in a basic spreadsheet.

### Characterization of methods performance

Serial dilutions of 5% MON 810 DNA were made corresponding to 40 000, 4000, 400, 200, 100, 20, 4 and 0.4 copies of invertase gene. For the MON 810 amplicon transgenic leaf material (100%) was used, with a 2:1 ratio between the invertase and the inserted construct. The same number of MON 810 copies was prepared as in the case of invertase detection, with the highest concentration at 8000 copies only. With higher copy numbers, inhibition was observed in all assays including TaqMan^®^. Higher copies were therefore excluded from systematic comparative studies. Each dilution was assayed in 5 parallels. The entire assay was subsequently repeated in an independent Q-PCR run. The same MON 810 DNA dilution series was prepared in EDTA buffer (Promega) for the Plexor™ assay, to ensure the required pH above 7.

For the purpose of this study the limit of detection (LOD) was set at the lowest DNA concentration with more than a half of positive parallels which corresponds to Results interpretation section of ISO 21569 standard [25]. To determine the lower limit of quantification (LOQ) a Cv ≤ 25% was the primary measurement parameter [26]. The only exceptions were assays with primer dimers detected in the dissociation stage. Those assays were assumed to be below LOQ when dimers contributed more than 1/3 to the signal. When below this limit, it was calculated they contribute less than 25% to the final copy estimation, which is inside our repeatability criteria.

To determine the dynamic range of the method and the PCR efficiency a standard curve was plotted of Ct values against the log of the estimated DNA copy number in the sample. The coefficient of determination (R^2^) was calculated and considered as suitable when not lower than 0.98 [27]. The slope of the standard curves (k) was used for efficiency calculation from the equation E = 10^[-1/slope]^-1 where an efficiency of 1 corresponds to 100% PCR efficiency [8]. The dynamic range of the method was then determined by LOQ at the lowest limit and at a dilution without inhibition at the highest. The PCR reaction was considered as not inhibited when the PCR efficiency was in the range of 75% ≤ E ≤ 120%. PCR efficiency within the dynamic range of the method should be in the range of 90% ≤ E ≤ 110% according to the recommendation of the European Network of GMO Laboratories (ENGL) [27].

The specificity of the methods for invertase was checked in two parallels with at least 1000 copies of target DNA in the reaction. For the specificity study on MON 810, two USDA samples were used with 0% of MON 810 GM maize: 1C 2003/5 (2.5% Bt11, 1% Bt176, 2.3% GA21, 0.5% T25, 1.1% NK603 and 0.1% CBH351) and C 2005/14 (0.06% Bt11, 0.03% TC1507, 1.3% T25 and 0.7% NK603). In terms of the DNA concentration, this indicates that at least 150 genome copies of each transgene maize tested were added per PCR reaction, except in the case of TC1507 and CBH351 maize, where only 7 and 24 DNA copies were present, respectively. In the case of amplification signal in these samples, isolated DNA from GM maize events NK603, GA21 and CBH351 obtained from Fluka (Buchs, Switzerland) and certified reference materials were used to additionally examine the cross-reactivity of the method.

Trueness of the methods was analyzed on two samples: 100% MON 810 leaf material (provided by CID-CSIC) on the upper range and 0.29% MON 810 maize flour (2C 2004/4 sample from USDA/GIPSA Proficiency Program) on the lower quantification range of the method. GMO amount was calculated as a ratio of transgene and endogene DNA copy numbers. In the same run the standard curve of 5% reference material was plotted and used for the calculation of the transgene content in the sample. For each amplicon three dilutions were made within the dynamic range of the method and run in two parallels. The GMO content was calculated for each of three pairs and the difference between obtained values was reported as repeatability. For 0.29% MON 810 sample data from the proficiency test were used and z-scores were calculated in the statistical environment of the R Project (base library) [28]. Measurement uncertainty is given as the range of 2 standard deviations.

The price to establish a new system was calculated on the basis of the price of all necessary chemicals to implement a new detection system, including the required master mix and minimal primer and probe concentrations needed to validate the system. The price of an individual reaction was calculated by assessing the costs of chemicals needed for one reaction with a 10 μl reaction volume. The labor intensity was evaluated by the number of hours required for the design and validation of a new method by trained personnel.

## Abbreviations

CPT: Cycling Probe Technology

Ct: cycle threshold (equivalent to Cp – crossing point)

Cv: coefficient of variation

FAM: 6-carboxyfluoresceine

GM: genetically modified

GMO: genetically modified organism

LNA^®^: Locked Nucleic Acid

LOD: limit of detection

LOQ: limit of quantification

Q-PCR: real-time quantitative PCR

RRS: Roundup Ready^® ^soybean

SD: standard deviation

TAMRA: 5-carboxytetramethylrhodamine

## Authors' contributions

MBG conducted laboratory experiments, analyzed the data and drafted the manuscript. KC helped with experimental work in the laboratory and participated in the design of the experiments. JŽ and KG participated in the design of the study, data analysis and drafting the manuscript. All authors read and approved the final manuscript.
